# 

**Published:** 1883-05

**Authors:** 


					﻿Contributors :
Alf. L. Looms, M.D.................New	York
Fessenden N, Otis, M.D............... “
F. H. Bosworth, M.D.................. “
J. L. Little, M.D.................... “
J. Williston Wright,	M.D.^,-......... “
C. E. Francis, D.D.S., M.D.S......... “
A. J. C. Skene, M.D..........Brooklyn,	N. Y.
C. A. Marvin, D.D.S..........
Edwin T Darby, M.D., D.D.S.......Phila..	Pa.
P. S. Wales, M.D.....Surg..-Gen’l	U. S. Navy
H. F. Campbell, M.D............Augusta,	Ga.
C. H. Mastin, M.D..LL.D.........Mobile,	Ala.
J. J. Chisolm, M.D,...........Baltimore,	Md.
C. F. Bevan, M.D.............. “
I.	E. Atkinson, M.D........... “
H. McGuire, M.D...............Richmond, Va.
F. P. Porcher, M.D..........Charleston,	S. C.
Jas. T. Whittaker, M.D.........Cincinnati, O.
J.	Ransohoff, M.D., F.R.C.S....	“
F. Forchheimeb, M.D................ “
A. R. Jackson, M.D...............Chicago, Ill.
S. V. Clevenger, M.D.............
E. F. INGALS, M.D.................. “
J. H. Carstens, M.D...........Detroit,	Mich.
PROSPECTUS
Fob Vol. IV., 1883, or the Independent
Pbactitionee.
This Jouenal is independent of colleges,
cliques, isms or any advertising firm; there-
fore impartiality pervades its columns in the
sole interest of the Profession.
It is cosmopolitan in its circulation and
scientific resources; consequently of equal
interest to the specialist and general prac-
titioner everywhere.
That which is new and useful in Medicine,
Surgery, Obstetrics, Dentistry, Pathology
and Popular Science is given to its readers
in an intelligent and concise style.
				

